# Simulation analysis of an adjusted gravity model for hospital admissions robust to incomplete data

**DOI:** 10.1186/s12874-023-02033-0

**Published:** 2023-09-29

**Authors:** Timo Latruwe, Marlies Van der Wee, Pieter Vanleenhove, Kwinten Michielsen, Sofie Verbrugge, Didier Colle

**Affiliations:** 1 Department of Information Technology, Ghent University, Technology Lane, Ghent, 9052 Belgium; 2 HICT, MeetDistrict, Ghent, 9000 Belgium

**Keywords:** Hospital admissions estimation, Gravity model, Healthcare planning, Huff Model

## Abstract

**Background:**

Gravity models are often hard to apply in practice due to their data-hungry nature. Standard implementations of gravity models require that data on each variable is available for each supply node. Since these model types are often applied in a competitive context, data availability of specific variables is commonly limited to a subset of supply nodes.

**Methods:**

This paper introduces a methodology that accommodates the use of variables for which data availability is incomplete, developed for a health care context, but more broadly applicable. The study uses simulated data to evaluate the performance of the proposed methodology in comparison with a conventional approach of dropping variables from the model.

**Results:**

It is shown that the proposed methodology is able to improve overall model accuracy compared to dropping variables from the model, and that model accuracy is considerably improved within the subset of supply nodes for which data is available, even when that availability is sparse.

**Conclusion:**

The proposed methodology is a viable approach to improve the performance of gravity models in a competitive health care context, where data availability is limited, and especially where a the supply nodes with complete data are most relevant for the practitioner.

**Supplementary Information:**

The online version contains supplementary material available at 10.1186/s12874-023-02033-0.

## Introduction

### Context

Facility location planning is commonly supported by gravity models. Gravity models identify a set of attraction poles, and based on attributes of the poles, an audience is attracted from a set of demand nodes. Attraction decays as geographical proximity decreases. By fitting gravity models to observed demand patterns, planners are able to derive rough rules on which demand volumes to expect, given a certain context. That context includes attributes of the pole, but also the competitive landscape the pole is located in, and attributes of demand nodes or even of the relationship between demand and supply nodes. A simple model might assert that the size of a supply node is what determines its attraction. Subsequently, this model might be fitted to empirical data of actual locations and market shares. As a result, the model would allow us to express the importance of size as a source of attraction and the amplitude of distance decay. Moreover, it could allow us to project derived patterns to, for instance, a hypothetical new supply node, yielding insight into its potential.

A common challenge in gravity modeling is feeding the models with data, especially if more sophisticated attraction variables are introduced. Imagine, for instance, the case of a retail chain considering to open a new store. The chain might have information on the location, size, and market share of all of the competitors in the region, and have additional details for its own stores, but lack this additional information for competing stores. Concretely, the chain could have carried out a survey to measure the reputation of its stores, but lack this variable for the competition. Therefore, the chain cannot introduce reputation as a factor of attraction in its modeling effort, because standard gravity models require this information for all attraction poles in the scope.

The challenge of incomplete data is addressed in this paper. First, a model is developed that supports distinctions between locations with and without values for particular attraction variables. Next, the performance of this model type is analyzed. Concretely, the accuracy of estimated effect sizes, i.e. the parameter coefficients, is reviewed as a function of the actual effect size, and the size of the sample for which complete data is available. Using simulated data with particular characteristics, it is possible to review results at various underlying conditions and draw conclusions on the meaning and value of estimates from this type of model.

The context of this paper is healthcare, specifically hospital facility planning. Therefore the scale, location, and number of facilities in our examples are set with this context in mind. Assumptions that have to be made in the course of the paper are likewise based on what is most appropriate in the hospital context. Nonetheless, the model could just as well be applied in a different context.

### Relevant literature

Limited data availability is a common challenge, since gravity models require quite extensive data availability. Several types of input data are commonly unavailable. First, the geographical origin of patients or clients is often missing. In those circumstances, distance decay coefficients cannot be empirically fitted. One approach is to assume them to be equal to coefficients found in other studies. Second, data for variables that could impact attraction is not always available or not always entirely available. In a gravity model analysis, entries for all supply nodes are required. It makes sense that in a competitive context, data might be available for some supply nodes and not for others, for reasons ranging from willingness to participate and share data, a lack of measurement in particular subgroups, to an inconsistent measurement methodology across groups. This work focuses on using data with the latter types of gaps.

In this paper, a gravity model is adjusted to be robust for missing data. Alternatively, aside from parameter assumptions, gaps in the data could be imputed. Widely used imputation techniques are mean imputation, median imputation, k-nearest neighbours (kNN) imputation, predictive mean matching (pmm) imputation, Bayesian Linear regression, non-Bayesian Linear regression, and Sample imputation methods [6]. Since it can introduce bias into the model, imputation requires careful consideration. For instance, mean or median imputation reduces the standard deviation of the considered variable, while regression imputation induces a linear relationship between attributes where the relationship might not be linear. The impact of missing data imputation has been reviewed by Brown and Kros [3] and Mishra and Khare [9], the latter analyzing different imputation methodologies using simulation. Generally data imputation is done when only few data points are missing. Jadhav et al. [6] advises a comparison of results before and after imputation if more than 25% of data is missing. The largest proportion of missing data that is worked with by Mishra and Khare [9] is 50%.

Comparing these approaches with the research described in this paper, two important distinctions should be highlighted aside from differences in methodology and model type. First, the authors are not only interested in the overall performance of parameter estimates for the full data sample, but also in the performance of estimates within a subgroup of supply nodes. Concretely, the subgroup for which data is not missing is studied separately because it often is the most relevant subgroup for practitioners. For instance, a network of hospitals, with exhaustive internal data, is likely even more interested in their own performance than in that of competitors. Second, this paper studies estimation accuracy in the context of more sparsely available data, with 5% up to 95% of data missing.

Data imputation is common in models that use large datasets and many variables. For gravity-type models in particular, little research work explicitly identifies data imputation in their methodology and none that the authors are aware of evaluates the impact of missing data. Shen and Aydin [10] is one exception that estimates missing cells in origin-destination matrices with a regression approach.

In a healthcare context, gravity models have been applied extensively [5], often with the objective to identify relevant catchment or service areas [7] or to evaluate accessibility of healthcare facilities [4, 11]. Not many studies explicitly discuss challenges related to data availability, and most commonly, effects of distance and size in terms of the number of beds are the only parameters that are estimated. This focus on accessibility exists partially because attraction by hospitals in a competitive context is often not the focus of a study, or less relevant depending on the healthcare system type.

### Objectives

The objectives in this paper are twofold. First, this paper validates or falsifies the efficacy of a gravity model type that distinguishes between locations based on the availability of data for those locations. Second, the paper describes the performance of this model type under various conditions, so that planners can better identify whether or not the model type would yield sufficiently good results in their context.

## Theoretical framework

In this section, the performed experiments are described. First, the model that accommodates missing data is specified. Second, we detail how estimation of the model is done. Third, the method for data generation is elaborated on. Lastly, the concrete experiments applied to the models are described.

### Model description

Generically, the model can be described as follows:

1 $$\begin{aligned} V_{ij} = \dfrac{ \left[ (1-B_j) + BM \times B_j \times \prod _{f=1}^{\mid \acute{F}\mid } {(\acute{A}_f^{\acute{\gamma }_{f}})}_{ij} \right] \times \prod _{f=1}^{\mid F\mid } {(A_f^{\gamma _{f}})}_{ij} \times e^{-D_{ij} \times DF} }{\sum _{j}^{J}{AA_{ij}}} \times V_{i} \end{aligned}$$

With

$$\begin{aligned} V_{ij} =& \text { Volume of demand for facility}\ {j}\ \text {in node}\ i. \\ B_j =& \text { Binary variable indicating whether facility}\ j\ \text {is part of the set for} \\ & \text { which all data is available.} \\ BM =& \text { Benchmarking parameter. } \\ | F | =& \text { Set of attraction variables } A_f \ \text { for which data is available for all facilities.} \\ | \acute{F} | =& \text { Set of attraction variables } \acute{A}_f \ \text { for which data is available for a fixed } \\ & \text { subset of facilities.} \\ A_f =& \text { Attraction variable part of set } | F |. \\ \acute{A}_f =& \text { Attraction variable part of set } | \acute{F} |. \\ \gamma _{f} =& \text {Parameter modulating the effects of variable } A_f. \\ \acute{\gamma }_{f} =& \text {Parameter modulating the effects of variable } \acute{A}_f. \\ D_{ij} =& \text { Great-circle distance between facility}\ j\ \text {and}\ i\ \text {in kilometers.} \\ DF =& \text { Parameter modulating the effects of variable } D_{ij}. \\ AA_{ij} ={} & {} \text { Result of the numerator for combination for facility}\ j\ \text {and node}\ i. \\ \end{aligned}$$

Equation (1) consists of several components. First the fraction on the right-hand side yields the estimated market share of facility *j* in node *i*. The market share, or the probability that a decision-maker in node *i* chooses facility *j* as a supplier, equals the perceived utility of *j* by the decision-maker in *i* divided by the sum of perceived utility of all alternative suppliers, expressed in a multiplicative utility function. The perceived utility function consists of two subcomponents: the utility-generating component and a distance decay component. The utility-generating component is the product of a set of variables $\mid F \mid$ that are expected to positively affect perceived utility. The distance decay component is a function of the proximity of *i* and *j*, in this case an exponential function.

In this particular model, a mechanism is added in order to accommodate the lack of complete data for a set of variables $\mid \acute{F} \mid$. Imagine, for instance, a regional study that yields values for a variable that is expected to affect perceived utility, such as reputation. The study does not cover the entire set of facilities that we want to study, but rather a subset $\mid \acute{F} \mid$. Since the numerator of the generic model described above is the sum of the utility of all alternatives, we would need a value for the reputation variable for all facilities in order to be able to apply it.

The model presented in Eq. (1) addresses this issue with two interventions. First, it introduces a binary variable $B_j$ to distinguish the utility function of facilities for which values for all variables are available from that of facilities without complete data. Second, it applies a parameter *BM* to the utility of hospitals for which all data is available to correct for the utility advantage that the extra variables bring. Since the variables are all positively correlated with utility, and are exclusively positive, any value, even a relatively low one, yields a comparative advantage with respect to facilities for which the variable is not included at all. The benchmark parameter *BM* should adopt a value such that it reflects (the inverse of) the average multiplication of utility that would be expected if values for the variables would be available. We choose to correct downwards the utility of facilities with full data rather than upwards the utility of facilities without full data because this implies that *BM* will take a value between 0 and 1.

In the example where reputation is the variable whose values are incomplete, we would expect that if reputation has a large effect on perceived utility, parameter *BM* would be low, because leveling the playing field given the absence of values for some facilities will require a more significant downward revision of their competitors’ utility. Implicit in this methodology is the assumption that the facilities whose reputation values are missing have an average reputation value, though specification of the average value is not required.

### Model estimation

The model presented in Eq. (1) cannot, as far as the authors know, be made linear in the parameters. The model is fitted without prior transformation using simulated annealing. The python package SciPy [13] is used, and 2000 iterations per fitting process are done before the solution with the minimal Mean of Squared Errors (MSE) is selected. After each round of annealing, local search with a maximum of 100 iterations is applied using the L-BFGS-B algorithm [14]. The error is defined as the difference between the observed number of demand for supply facility *j* and the estimated number given the parameter instances.

### Data simulation

A data simulator was designed for the purpose of this research. In order to generate datasets with particular characteristics, five steps are followed. First, (1) a dataset is given that represents the facility and demand nodes (areas) that data will be generated for. Next, (2) random instances are generated for the variables under consideration. Subsequently, (3) utility per facility is calculated based on the generated variables and their parameters, which is (4) translated into market shares or choice probabilities. Lastly, (5) discrete demand is generated according to the choice probabilities.

The initial dataset provided to the generator (1) contains the locations of, and distance between, all supply and demand nodes. It thus has $J \times I$ rows, with *J* the total number of facilities *j*, and *I* the number of areas *i*. In addition, it contains total market size per area *i*. Using pre-set configurations, (2) values are drawn from probability distributions that reflect expected values per attraction variable in the model. Values can be set on the supply-node level, if the factor describes a characteristic of the node, or on a combination of supply node and demand node, if the factor describes a characteristic of the relationship between the two, such as localized reputation. A generator for the value can be given, supporting the use of various probability distributions. For the reputation variable $REP_J$, a normal distribution with mean 10 and standard deviation 2.8 is used.

At this point in the process, values for all of the attraction variables in the model are available. The exponent of each attraction variable, signifying effect size, is made available in the pre-set configuration. The exponent can be based on actual measurements, or be set arbitrarily as required to perform the experiments in this paper. Given this input, the perceived utility of each facility *j* for each demand area *i* according to the multiplicative utility function can be computed (3). The translation of utilities into market shares or choice probabilities is the same as the share of a facility’s utility in the utilities of all of the alternative supply nodes for an area (4).

Subsequently (5), the set of choice probabilities for all facilities per area *i*, which together form a multinomial probability distribution, are used to generate discrete demand volumes. Concretely, the Python function *choices* from the package random is used, with the array of probabilities as weights, and the number of demand units for the full area *i* as *k*.

### Experiments

The concrete experiments performed in this research are modeled on the scale and structure of hospital facilities in the Flemish market. The locations of supply nodes used are derived from the locations of hospitals in Flanders, excluding Brussels. This corresponds with 89 campuses or facilities across the geographical area. The locations of the demand nodes or areas *i* are the centroids of each geographical unit. In total, 9139 units are used, as delimited by STATBEL [12], and corresponding to the Flanders area except Brussels. The size attraction factor is also set to the actual size quantified as the number of recognized beds of each facility. The reputation factors and observed demand are generated by the process described in section “Data simulation”. For each of the following experiments, size and reputation are included as attraction variables. The exponent of size is set at 0.92, which is a realistic value for this sample and context, as derived in other work [8]. Reputation will be the randomly selected factor. Its exponent will be varied between 0.1 and 1.3. The number of facilities for which reputation information is available is varied between 5 and 100%. For each of these combinations, five runs are executed, which yields 700 runs in total.

A couple of results are expected. First, the accuracy of reputation exponent estimates is expected to improve as the number of facilities for which reputation is available increases. Second, it is expected that the relative accuracy will increase as the effect size increases.

With each experiment, the sources of variation are limited as much as possible in order to limit noise. The reputation values are generated only once, and reused per variation in exponent or number of facilities with full information. For variations in the number of facilities with data available while keeping the effect size equal, the patient volumes can be kept identical since they are unaffected by that type of variation.

## Results

**Fig. 1:**
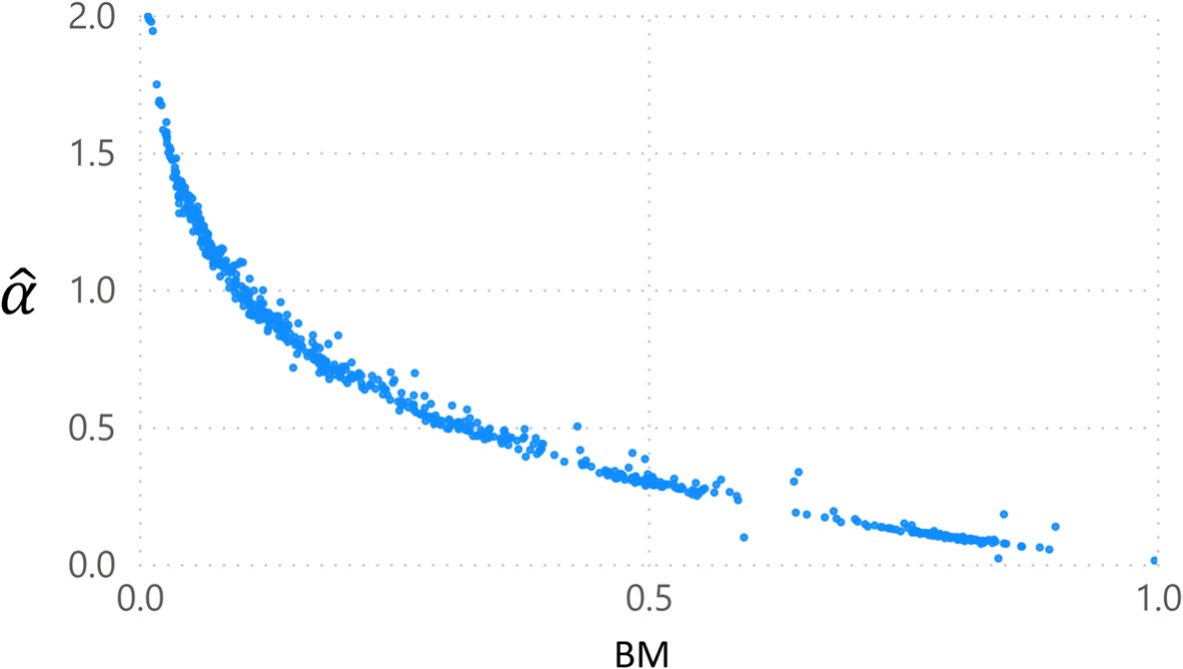
The graph shows the relationship between $\alpha$ and the balancing parameter *BM* across all experiments: 5 runs for 7 effect sizes, and 19 sample sizes (increments of 0.05 starting at 0.05 up to 0.95), yielding 665 observations

First, the balancing mechanism of the model is evaluated. Figure 1 shows the relationship between $\alpha$, the coefficient of the reputation parameter, and *BM*. As intended, the two are closely related. The *BM* parameter scales down the utility for facilities that lack reputation information. Accordingly, it is strongly related to the strength of the impact of reputation, as measured by $\alpha$. Given that the relationship between $\alpha$ and *BM* should theoretically be perfect, it should be possible to write *BM* as a function of $\alpha$ and remove it as a parameter that needs to be estimated. This paper does not explore this avenue further.

**Fig. 2:**
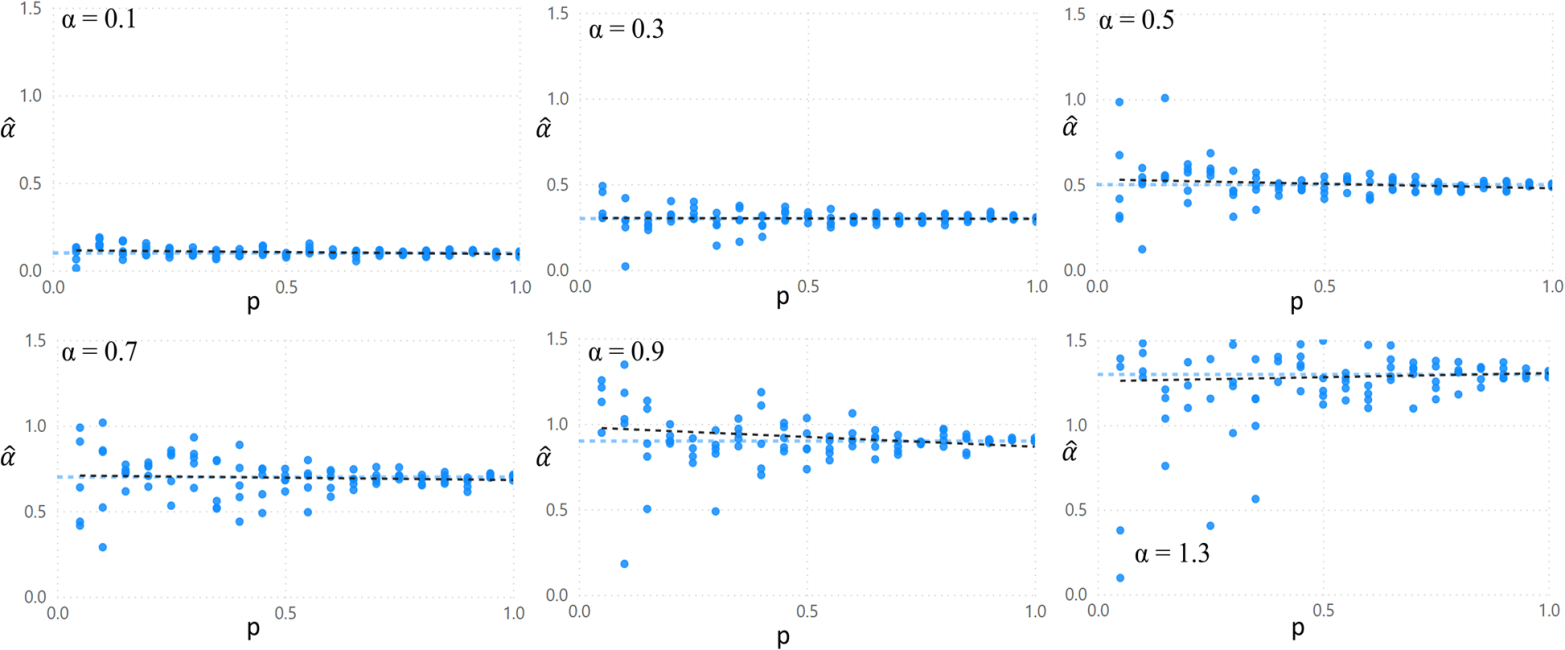
For different effect sizes, the figure shows the estimated effect size by the model. P is the proportion of facilities for which reputation data is made available to the model

Next, the effect of the proportion of facilities for which reputation information is available is evaluated. Figure 2 shows the results of parameter estimates for $\alpha$, plotted against data availability. It is observed that a tendency exists for the accuracy of estimates to improve as the proportion of facilities for which reputation data is available is increased. For each combination of *p* and effect size, three observations are available.

The Breusch-Pagan test [2] is used to evaluate whether the error variances are a multiplicative function of *p*, which represents the proportion of the data that is not missing. The *p*-value of the F-statistic does not yield a statistically significant value that would confirm the expected heteroskedastic pattern in the residuals.

**Fig. 3:**
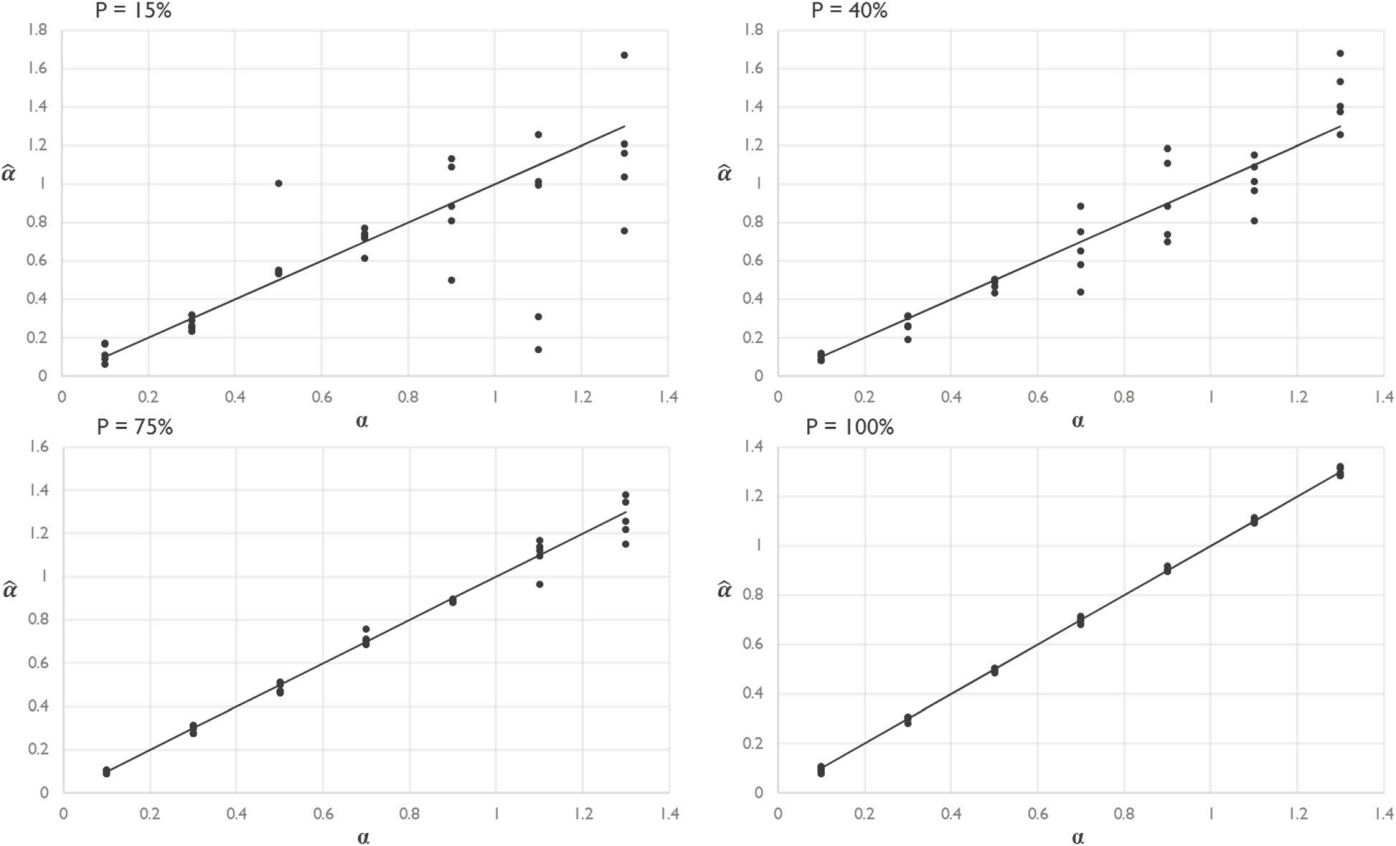
The figure shows four scatterplots of different data availability scenarios that compare the estimated effect size of the reputation parameter and its value as introduced in the generated dataset

In addition, the impact of effect size on estimation accuracy is reviewed. Figure 3 suggests a lower absolute estimation accuracy at higher effect sizes.

**Fig. 4:**
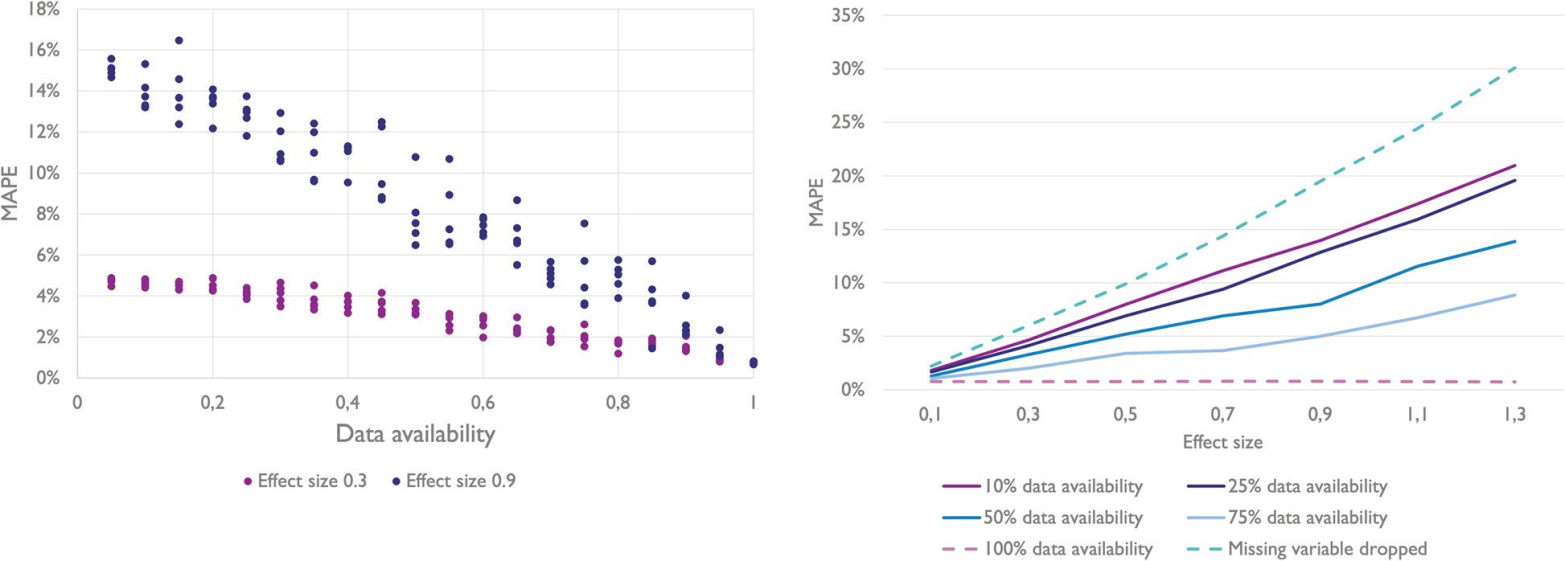
The figure on the left shows the relationship between the most important error metric, the Mean Average Percentage Error (MAPE), and data availability for effect sizes 0.9 and 0.3. The figure on the right shows the average MAPE for different data availabilities plotted against the effect size

Aside from evaluating the efficacy of estimating the reputation parameter, the overall performance of the model is assessed. Figure 4 shows that a clear relationship exists between model accuracy and reputation sample size. As expected, the effect size does not systematically affect the model accuracy when reputation data is available for each point. When only limited data is available, however, effect size positively correlates with the error metrics, reflecting the conditions that were introduced into the dataset. Concretely, the reputation effects at work in the cases where the reputation variable is not available are a cause of unmodelled variation in the data. Two boundary scenarios are shown, along with scenarios in which the degree of data availability differs in between. The *control* boundary scenario is one in which the reputation parameter is left out of the model, it is the result obtained when fitting a classic gravity model that uses the facility size parameter as an attraction factor, and none other. The *100%* boundary scenario is one in which there is full data availability, meaning that reputation data points are available for each facility. These scenarios show how the overall Mean Average Percentage Error (MAPE) generally improves as more reputation data points are available and used.

**Fig. 5:**
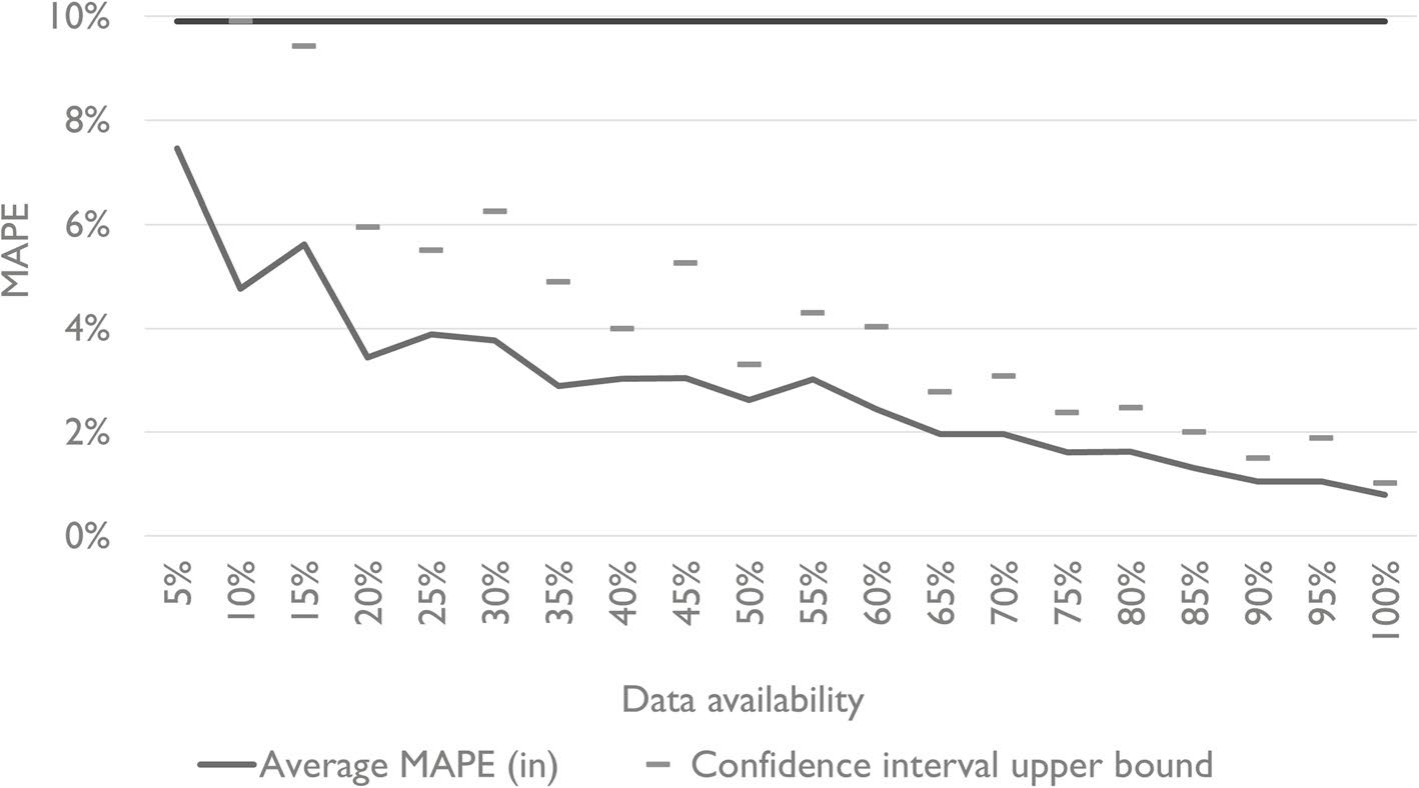
The figure shows the in-group MAPE for different reputation data availability levels given an effect size of 0.5. A right-tailed student’s T distribution with 4 degrees of freedom was used to calculate the t-value=2.13, on the 5% significance level. The upper bound for $p=0.05$ is 0.20, which is out of the range of the y-axis

An interesting and perhaps more crucial question is how the model has performed within the group of facilities for which the additional data was available. After all, the primary user of this model type is likely most interested in this scope. Table 1 shows that the MAPE is markedly better for facilities for which the reputation variable is available and used. Given a limited reputation data availability of 25%, and a relatively strong effect size of 0.7, the in-group estimates have a MAPE of 5.6%, while the out-group estimates have a MAPE of 13.2%. The latter is close to the accuracy achieved when not using the reputation variable in the model at all (14.4%). This observation is consistent across effect sizes and data availability. The out-group estimates are about as accurate as those of a model that does not use the reputation variable. Even for cases with low data availability, improvements of in-group accuracy can be considerable, as shown in Fig. 5.

Nonetheless, the variance of the MAPE is relatively high, especially in cases where the number of observations of the variables with missing values is small. Despite that, the upper bounds of the 95% confidence intervals of the MAPE for the in-group with sample size above 10% is consistently lower than the MAPE measured when discarding the variable.

**Table 1:** Shows demand estimation accuracy in subgroups of the analysis. Concretely, the MAPE on the facility level is measured for the group of facilities that has data available for the reputation variable, and for the group that does not

	Proportion of facilities with available reputation data							
	25%		50%		75%		100%	0%
Effect size	MAPE (in)	MAPE (out)	MAPE (in)	MAPE (out)	MAPE (in)	MAPE (out)	MAPE	MAPE
0.1	1.1%	2.2%	1.0%	2.1%	1.0%	2.1%	0.8%	2.2%
0.3	1.9%	5.9%	1.4%	6.0%	1.4%	5.7%	0.8%	6.0%
0.5	3.9%	9.8%	2.6%	9.4%	1.6%	9.0%	0.8%	9.9%
0.7	5.6%	13.2%	3.7%	12.8%	2.2%	13.3%	0.9%	14.4%
0.9	6.5%	18.6%	4.1%	16.9%	2.4%	18.4%	0.8%	19.5%
1.1	10.9%	22.6%	5.2%	23.5%	3.2%	23.5%	0.8%	24.4%
1.3	9.7%	27.2%	6.7%	30.5%	5.1%	28.7%	0.8%	30.1%

## Discussion

The experiments in this research show that it is possible and can be effective to include variables in gravity models for which only limited data is available. The evidence the experiments provide is generated under particular, though broadly relevant, conditions. First and foremost, the input data is simulated in a process that produces the type of structure that a gravity model is meant to reflect. Despite empirical successes, gravity models have often been criticized for lacking sound theoretical underpinnings [1]. Correspondingly, there is no strong theoretical basis to justify the assertion that the generated data should adopt this structure. Second, due to limited resources, the behaviour and performance of the model has not been evaluated in all possible circumstances. Concretely, relevant circumstances that are not studied might be larger numbers of nodes, significant differences in the ratio of demand and supply nodes and related average proximity, the use of more explanatory variables in the model, or the use of different probability distributions for the explanatory variables.

Within these bounds, this research shows that significant improvements in model performance can be achieved. If a variable is dropped because of incomplete data, a loss of accuracy occurs that is plainly and strongly related to the effect size of the variable. In contrast, including even sparsely available data using the described methodology improves the accuracy of the model markedly for those supply nodes for which data is available without adversely affecting accuracy for supply nodes that lack the relevant data. Accordingly, this evidence suggests that adding sparsely available data in gravity models can provide incremental improvements for variables with a small impact to sharp improvements for those with a high impact. On average in cases of sparsely available (25%) data, the improvement of the MAPE is 61.3% for the in-group as compared to a model that excludes the incomplete variable.

## Future work

As this research shows, it is possible to improve the results of a gravity model by including variables for which data is incomplete with adjustments of the model specification. Nonetheless, it has not been evaluated how this methodology compares to data imputation techniques. An interesting avenue for further research is clarifying the relationship between the proposed methodology and data imputation techniques, along with the evaluation of their comparative performance. A general gap in data imputation literature is the evaluation of performance within the subgroup for which data is available. In the context of this specific paper, gaps in the data are not random or coincidental, and accordingly, subgroup performance has a specific and relevant meaning. A comparison with imputation techniques should review this dimension of performance specifically.

### Supplementary Information

**Additional file 1.**

## Data Availability

All data generated or analysed during this study are included in this published article and its supplementary information files.
