# Supplementary material for: Simulation analysis of an adjusted gravity model for hospital admissions robust to incomplete data
Source: BMC Med Res Methodol. 2023 Sep 29;23:215. doi: 10.1186/s12874-023-02033-0 (PMC10540423; doi:10.1186/s12874-023-02033-0)
Supplement: Supplementary file 1 — Additional file 1. [file 12874_2023_2033_MOESM1_ESM.pdf]

LEGEND

file 1: *base_layer_dataset-gravity-repl.csv*
i: The area code of individual statistical sectors in the relevant Geographic region
j: The campus code
D_ij: Distance in kilometers between the center of area i and campus j.
S_j: Size in terms of recognized beds in campus j
PDMV_i: Number of admissions in area i

file 2: *Results Dataset.csv*
index: index number
DV: parameter value related to distance in gravity model
BETA: Parameter value related to the size parameter
ALPHA: Parameter value related to the impact of the reputation parameter
BM: Benchmark parameter value
effect_size: the effect size induced in the dataset
p: the sample rate of observations of the reputation variable
label: label for the model structure
MAPE_AGR: mean average percentage error measured on the hospital campus level
WMAPE_AGR: weighted mean average percentage error measured on the hospital campus level, weighted by the volume of observed admissions
LS_AGR: Least Squares error metric measured on the hospital level
LS_MUNI: Least Squares error metric measured on the municipal level
IN_MAPE_AGR: Mean average percentage error measured on the hospital campus level for the group of hospital campuses that have observations of the reputation variable
IN_WMAPE_AGR: Weighted Mean average percentage error measured on the hospital campus level for the group of hospital campuses that have observations of the reputation variable, weighted by the volume of observed admissions
IN_LS_AGR: Least Squares error metric measured on the hospital campus level for the group of hospital campuses that have observations of the reputation variable
OUT_MAPE_AGR: Weighted Mean average percentage error measured on the hospital campus level for the group of hospital campuses that do not have observations of the reputation variable, weighted by the volume of observed admissions
OUT_LS_AGR: Least Squares error metric measured on the hospital campus level for the group of hospital campuses that do not have observations of the reputation variable
OUT_LS_MUNI: Least Squares error metric measured on the municipal level for the group of hospital campuses that do not have observations of the reputation variable

FILE: GravityGenerator.py

from functools import cached_property

import numpy as np

import random

class GravityGenerator:

"""

A class that generates a modified DataFrame based on the input DataFrame and factor configurations in factor_configs.

This class is used to calculate the patient volume for each origin-destination (i, j) pair

in the input DataFrame. It takes into account the factors specified in the factor_configs parameter.

Each factor configuration should include the label, name, exponent_name, exponent_value, level, and

generator function for the factor. The GravityGenerator class also considers the distance

factor when calculating the weighted patient volume.

Attributes

----

input_df : pd.DataFrame

The input DataFrame containing information about origin-destination (i, j) pairs. It must contain a column i, and j, with identifiers for the origin and destination, respectively. It must also contain a column D_ij, with the distance between the origin and destination.

factor_configs : list

A list of dictionaries, where each dictionary represents a factor that affects the patient volume in the Gravity Generator model. These factors are used to calculate the weighted patient volume for each origin-destination (i, j) pair in the input DataFrame. Each dictionary in the factor_configs list should contain the following keys:

"label" (string): A human-readable label for the factor.

"name" (string): The name of the column in the DataFrame that will contain the generated factor values.

"exponent_name" (string): The name of the exponent associated with the factor.

"exponent_value" (float): The value of the exponent used in the calculations.

"level" (string): Indicates the aggregation level of the factor. It can be one of the following options:

"i": The factor is aggregated at the origin level.

"j": The factor is aggregated at the destination level.

"ij": The factor is aggregated at the origin-destination level.

"generator" (function): A function that generates the factor values when applied to the DataFrame. For instance, def generator(): return np.random.normal(0, 1).

_distance_factor : float, optional, default=0.16

The distance factor to be considered when calculating the weighted patient volume.

volume_name : str, optional, default="V_ij"

The name of the column in the output DataFrame containing the weighted patient volumes.

Methods

_df_wt_generated_factors():

Returns a modified DataFrame with additional columns of factors generated according to the given factor_configs. The method adds new columns to the DataFrame based on the provided factor_configs, using the specified generator function and the level in each factor_config.

_attr_factor_configs():

Filters and returns the factor_configs that contain the "exponent_value" key.

```
    _df_wt_generated_volumes(df=None):

        Returns a DataFrame containing the discrete patient volumes for each origin-destination (i, j) pair. This function computes the weighted patient volume for each pair based on the provided factors and distance factor and returns the modified DataFrame with the new values.

    transform_probabilities_to_v_ij(x):

        Transforms the probabilities of a given DataFrame into the corresponding V_ij values by sampling from the probability distribution. This method is used during the calculation of the discrete patient volumes for each origin-destination (i, j) pair.

    """

    def __init__(self, *args, input_df, factor_configs=None, distance_factor=0.16, volume_name="V_ij"):

        self.input_df = input_df.reset_index()

        self.factor_configs = factor_configs

        self._distance_factor = distance_factor

        self.volume_name = volume_name

    @cached_property
    def _df_wt_generated_factors(self):
        """

        Generate a DataFrame with additional columns of factors generated according to the given factor_configs.

        This method takes the input DataFrame, iterates through the factor_configs, and generates a new column

        for each factor_config using the specified generator function. The generator function is applied to the

        group defined by the level in the factor_config. The modified DataFrame is then returned.



        Returns:

        -------
```

```
        df : pd.DataFrame

            The input DataFrame with additional columns of generated factors.

        """

        # Step 1: Get the input DataFrame

        df = self.input_df

        # Step 2: Iterate through the factor_configs

        for i, factor_config in enumerate(self.factor_configs):

            # Step 3: Generate the new factor column using the generator function and the
specified level

            df[factor_config["name"]] = df.groupby(list(factor_config["level"]))[
                "j"].transform(lambda x: factor_config["generator"]())

        # Step 4: Return the modified DataFrame

        return df

    @property
    def _attr_factor_configs(self):
        """

        Filter the factor_configs to return only those that contain the "exponent_value" key

        Those are the 'attraction factors'

        """

        return list(filter(lambda x: "exponent_value" in x, self.factor_configs))

    def _df_wt_generated_volumes(self, df=None):
        """

        Compute the discrete patient volumes for each origin-destination (i, j) pair in a
DataFrame.

        This function takes a DataFrame containing information about origin-destination (i, j)
pairs and
```

```
        factors affecting the patient volume. It computes the weighted patient volume for each
pair and

        returns the modified DataFrame with the new values.


        Parameters

        ----------

        df : pd.DataFrame, optional

            DataFrame containing information about origin-destination pairs and factors affecting

            the patient volume. If not provided, the function will use the DataFrame stored in the

            _df_wt_generated_factors attribute of the object.


        Returns

        -------

        df : pd.DataFrame

            DataFrame containing the discrete patient volumes for each origin-destination (i, j)
pair.

        """

        # Check if a DataFrame is provided, otherwise use the stored DataFrame

        if df is None:

            df = self._df_wt_generated_factors

        # Set the DataFrame index to origin-destination (i, j) pairs

        df.set_index(["i", "j"], inplace=True)

        # Calculate utility U_ij using the given factors and distance

        df["U_ij"] = (

            np.prod(
```

```
            [
                np.power(df[factor_config["name"]],
                         factor_config["exponent_value"])
                for factor_config in self._attr_factor_configs
            ],
            axis=0,
        )
        / np.exp(df["D_ij"].values * self._distance_factor)
    )
    # Calculate the sum of U_ij values for each origin (i)
    df["AU_i"] = df.groupby("i")["U_ij"].transform("sum")
    # Compute the probability P_ij of choosing j in area i for each origin-destination (i, j) pair
    df["P_ij"] = df["U_ij"].values / df["AU_i"].values
    # Calculate the patient volume for each origin-destination (i, j) pair
    df[self.volume_name] = (
        df.groupby("i")[["P_ij", self.volume_name[:-1]]].apply(
            lambda x: self.transform_probabilities_to_v_ij(x).to_frame("n")
        ).values
    )
    return df
def transform_probabilities_to_v_ij(self, x):
    """
    Transforms the probabilities of a given DataFrame into the corresponding V_ij values by
    sampling from the probability distribution.
```

```
        Parameters:
        x (pd.DataFrame): The input DataFrame containing probabilities and volume.


        Returns:
        pd.Series: The modified DataFrame column containing the V_ij values.
        """
        # Extract the volume value from the input DataFrame and round it
        volume = round(float(x[self.volume_name[:-1]].values[0]))
        # Sample indices based on the probabilities and volume
        indices, _, counts = np.unique(random.choices(
            range(len(x)), x["P_ij"],
            k=volume), return_index=True, return_counts=True)
        # Initialize an array of zeros with the same length as the number of rows in the input DataFrame
        a = np.zeros((len(x.index),))
        # If volume is greater than 0, place the counts at the corresponding sampled indices in the array
        if volume > 0:
            np.put(a, indices, counts)
        # Update the input DataFrame with the new V_ij values
        x[self.volume_name] = a
        # Return the modified V_ij column from the input DataFrame
        return x[self.volume_name]
```
